# Improving disease classification performance and explainability of deep learning models in radiology with heatmap generators

**DOI:** 10.3389/fradi.2022.991683

**Published:** 2022-10-11

**Authors:** Akino Watanabe, Sara Ketabi, Khashayar Namdar, Farzad Khalvati

**Affiliations:** ^1^ Engineering Science, University of Toronto, Toronto, ON, Canada; ^2^ Department of Diagnostic Imaging, Neurosciences / Mental Health Research Program, The Hospital for Sick Children, Toronto, ON, Canada; ^3^ Department of Mechanical and Industrial Engineering, University of Toronto, Toronto, ON, Canada; ^4^ Institute of Medical Science, University of Toronto, Toronto, ON, Canada; ^5^ Vector Institute, Toronto, ON, Canada; ^6^ Department of Medical Imaging, University of Toronto, Toronto, ON, Canada; ^7^ Department of Computer Science, University of Toronto, Toronto, ON, Canada

**Keywords:** heatmaps, disease classification, radiology, explainability, deep learning, medical imaging

## Abstract

As deep learning is widely used in the radiology field, the explainability of Artificial Intelligence (AI) models is becoming increasingly essential to gain clinicians’ trust when using the models for diagnosis. In this research, three experiment sets were conducted with a U-Net architecture to improve the disease classification performance while enhancing the heatmaps corresponding to the model's focus through incorporating heatmap generators during training. All experiments used the dataset that contained chest radiographs, associated labels from one of the three conditions [“normal”, “congestive heart failure (CHF)”, and “pneumonia”], and numerical information regarding a radiologist's eye-gaze coordinates on the images. The paper that introduced this dataset developed a U-Net model, which was treated as the baseline model for this research, to show how the eye-gaze data can be used in multi-modal training for explainability improvement and disease classification. To compare the classification performances among this research's three experiment sets and the baseline model, the 95% confidence intervals (CI) of the area under the receiver operating characteristic curve (AUC) were measured. The best method achieved an AUC of 0.913 with a 95% CI of [0.860, 0.966]. “Pneumonia” and “CHF” classes, which the baseline model struggled the most to classify, had the greatest improvements, resulting in AUCs of 0.859 with a 95% CI of [0.732, 0.957] and 0.962 with a 95% CI of [0.933, 0.989], respectively. The decoder of the U-Net for the best-performing proposed method generated heatmaps that highlight the determining image parts in model classifications. These predicted heatmaps, which can be used for the explainability of the model, also improved to align well with the radiologist's eye-gaze data. Hence, this work showed that incorporating heatmap generators and eye-gaze information into training can simultaneously improve disease classification and provide explainable visuals that align well with how the radiologist viewed the chest radiographs when making diagnosis.

## Introduction

Complex deep learning models have been more recently incorporated into clinical radiology practices and have given promising results to assist radiologists in identifying and classifying various diseases and abnormalities, such as pneumonia and congestive heart failure (CHF) (1). Nevertheless, it is quite difficult for humans, including radiologists, to understand how such deep learning models arrived at their predictions (2). Unlike linear regression or support vector machines that have fewer-dimensional classification boundaries, which are easier to understand and visualize, deep learning algorithms are often referred to as black-box algorithms because of their computational complexity and the fact that we are often unable to easily observe the decision boundaries generated by those models (1–4). In fact, performance and explainability have often been traded-off, and models with better performance tend to be worse in terms of explainability and vice versa (3, 5, 6).

Hence, the explainability of Artificial Intelligence (AI) models that shows the part of the model's inputs or the kind of information the models focused on when making predictions is crucial for the radiology field to gain and increase radiologists', patients', and regulators' trust in the use of the models for diagnosis (2, 5). The explainability aspect of the models also helps to verify the model's conclusions and to identify models' biases (6–8). A model with improved classification and enhanced explainability components will be able to assist the radiologists by making the diagnosis process more efficient and minimizing the risk of making mistakes (7). With the increase in explainability, the model can also be widely used at medical facilities that perform Chest x-Ray (CXR) imaging where there may not be enough radiological expertise to identify and classify diseases.

The methods to establish the explainability of the models can involve different data types, such as mathematical computations, texts, or visualization with heatmaps or saliency maps (1). In the radiology field, CXR images are one of the essential components for diagnosing abnormalities or conditions that affect chest and nearby organs, and many AI models for radiology often use the chest radiographs as one of their inputs (7, 9). Thus, the visualization aspect of explainability on images with heatmaps is an impactful tool to convey which part of the radiograph the model observed closely for illness classification (1, 10).

Currently, there are several studies on using deep learning models for disease classification that also involve heatmap generation to visualize where the model's focus was on the given input CXR image (9, 11). Nonetheless, there are many limitations in such research. For example, many publicly available datasets that such studies use contain erroneous class labels because the labels were often extracted from text reports using natural language processing (NLP) models (9, 12), and the state-of-the-art NLP models still cannot achieve 100% accuracy on texts interpretation. Additionally, many of the models use only CXR images and disease class labels for training, but many diseases can only be classified through using other information the CXR images cannot necessarily provide, such as symptoms, clinical signs, patients' history, and results from blood tests (9). Lastly, many of the studied models do not consider the methods the radiologists usually take to analyze the CXR images, such as the way they view the chest radiographs to make diagnosis (12). It has been shown that integrating eye-gaze information improves AI models' classification performance and has been validated that eye-gaze data contains valuable information related to focusing on important input features (13).

To increase the classification performance and the explainability of AI models while mitigating such dataset and labeling limitations, this work proposes and tests three different methods that use a U-Net-based (14) model. The methods verify whether incorporating various gradient-based heatmap generators, such as guided back-propagation, in addition to radiologists' eye-gaze information, CXR images and class labels into the model training can improve disease classification while enhancing explainability. While utilizing U-Net for generating heatmaps is not novel, the application of the gradient-based heatmap generators in addition to the radiologist's eye-gaze data during model training has not been fully studied before. This research used a dataset (12) that contains not only the CXR images and corresponding class labels (which are “normal”, “CHF”, and “pneumonia”), but also a radiologist's eye-gaze coordinates information received when the radiologist was viewing the CXR image to perform diagnosis. The focus of this paper is on improving the classification performance, and hence, no quantitative approach is employed for assessing explainability, which is a limitation to be addressed in future works.

Although the proposed models, mostly had similar overall average classification performances as the baseline model that did not use heatmap generators in the training process, there were greater improvements in classifying the “CHF” and “pneumonia” classes, which were the classes the baseline model struggled to correctly classify. Moreover, one of the proposed models had a superior improvement in both the overall average classification performance and the performances on “CHF” and “pneumonia” classes.

The followings are the key contributions of this research:

- This research introduces three experiment sets that uniquely use gradient-based heatmap generators, such as deconvNet, and a radiologist's eye-gaze data during models' training.
- This study shows the experiment sets improved the classification performance of the AI model, especially for “pneumonia” and “CHF” classes. Hence, the proposed model was able to decrease the number of false negatives with the use of heatmap generators and eye-gaze data.
- This research conveys that the use of the heatmap generators and eye-gaze data can further enhance the explainability of the model by training the model to observe the input images in a similar manner to how the radiologist views them. Such enhanced explainability for the improved classifier can increase trust in the use of these AI models among clinicians and patients.

## Background

This section reviews the notion of the “explainability of AI” used in this study. Furthermore, two prominent paths of incorporating explainability into AI for radiology are specified, which include visualizing with saliency maps and training deep learning models to directly generate attention maps as one of their outputs. In this research we only consider spatial explainability because it is correlated with the eye-tracking data available. Thus, the literature review is focused on the spatial explainability of AI models.

### Explainability of AI in radiology

The concept of “explainability of AI” is often defined as the ability of someone to understand and see which extracted features of the input data used by an AI model contributed to the model's predictions (1, 2, 5). It also helps to identify models' biases and understand how the model achieved its predictions (7). Thus, methods for explainability of AI should ensure that models did not operate on unrelated features (15).

There are two major key methods to showcase the explainability aspects of AI models for the radiology field when using chest radiographs as a part of a model's training. Firstly, various saliency maps (also known as attention-maps, heatmaps, or sensitivity maps) generation methods can be incorporated within or after training a deep learning model to highlight the areas of the input chest radiographs the model's parameters focused on when making predictions. Secondly, there are deep learning models that were developed to simultaneously operate disease classification and heatmap generation using image segmentation.

#### Various saliency maps and attention map generators

Saliency maps show the parts of the input image that contributed most to the model's output predictions (16). Some of the post-hoc, gradient-based methods that can be used to obtain the saliency maps are Gradient-weighted Class Activation Mapping (Grad-CAM) (17), deconvNet (18), back-propagation, guided back-propagation (GBP) (7, 19), and class activation maps (CAM) (20). Each of the generators produces visually different heatmaps that highlight the section of the image that corresponds to the class that a model predicted. All of them are variations of deconvolution and back-propagation methods (21), where deconvolution attempts to recreate the input image from the activations of the model's layer, while back-propagation is how a model's weights change during training time to decrease its loss. Back-propagation is used to find the relevance of the input pixels to the output predictions based on how the gradients were assigned to those pixels (19). The generated heatmaps are the visualization of such computations or gradients. Gradients with a larger magnitude signify that the corresponding pixels have more influence on the specific classification of the image.

Procedures for deconvNets and GBP are quite similar where the difference between them is the way each backpropagates through ReLU non-linearity. DeconvNets run the usual back-propagation by using transpose convolution and undoing pooling operations, and they back-propagate only the positive error signals for the ReLU activation (19).

GBP maps are produced through the combination of the deconvolution and back-propagation methods. The deconvolution part shows which pixels contributed positively to the model's output through selectively back-propagating the positive component of the gradients between the input and the output of the models; meanwhile the back-propagation part restricts the model to consider only the positive inputs, which can result in GBP maps having more zeros in the outputs than DeconvNets (18), and hence, GBP maps tend to have higher resolution.

CAM (20) is another visualization map, which is produced using a global average pooling layer in CNN models. CAM's pooling layer reduces each feature map into one number, so that the weights connecting the pooling layer and the final classification layer encodes the contribution and the importance of each feature map to the final class prediction.

There are several modifications and improvements made on CAM for image analysis and computer vision tasks. For example, Pyramid Localization Network, also known as PYLON, (15) was developed to produce higher resolution of CAM heatmaps with greater preciseness using Pyramid Attention mechanism and upsampling blocks.

Because CAM can only be produced using a specific set of CNN models, such as those without fully connected layers, Grad-CAM (17) was proposed as a generalization of CAM to eliminate the necessity of a trade-off between model accuracy and explainability and to avoid model retraining (1). It can be applied to many variations of CNN models, including those with fully connected layers (1, 3, 17). Grad-CAM focuses on the gradients flowing into the last convolutional layer of the model and assigns importance scores to each neuron to generate localization maps. The importance score, which is the contribution of a specific feature map to the model's output, is computed by finding the gradient values for a specific class with respect to the activation map of a convolutional layer and using global average pooling on the gradients. This results in a coarse heatmap that is the same size as the convolutional feature map, and grad-CAM will only consider features that have a positive influence on the specific class.

There are multiple variations of grad-CAM, including guided grad-CAM (22) and GradCAM++ (22). Guided grad-CAM is an element-wise multiplication of GBP and grad-CAM, which results in higher resolution, class-discriminative maps. GradCAM++ focuses on the weighted average of the positive partial derivatives of the last convolutional layer's feature maps with respect to a specific class for better object localization and for recognizing multiple class objects in a single image.

SmoothGrad (23) is a method that reduces noise in the output saliency maps. This method first adds various noise into an input image and then produces look-alike images. Finally, the method uses those images to generate many saliency maps that can be averaged to produce one saliency map with less noise. It is addressed in response to the possibility that the noise in the saliency maps is due to irrelevant, local fluctuations in partial derivatives. It has been shown that combining smoothGrad and GBP methods can produce more visually coherent maps (23).

DeepLIFT (Deep Learning Important FeaTures) (24) also uses back-propagation method for explainability. Specifically, DeepLIFT employs “difference from reference” concept for neurons' activations to determine importance scores for each input through back-propagating the model once.

Unlike the use of gradient-methods and back-propagation related methods for explainability, LIME (Local Interpretable Model-Agnostic Explanations) (25) creates an interpretable model through approximating the explanations locally around classifiers and providing a method that chooses several combinations of example inputs and corresponding explanations to address why the model should be trusted. LIME can give explanations to model predictions for any classifier or regressor.

Although there is much debate about the true effectiveness of saliency map generation, the output maps give insights into how a model arrives at its prediction output, and the maps are often used post-training to visualize the model's attention on test data.

### Deep learning models as heatmap generators and classifiers

As opposed to the above heatmap generating methods that can be inserted during or after training AI models, there are several studies involving constructing deep learning models or pipelines that focus on generating heatmaps as part of the model's outputs and on operating localization tasks. One of the more widely used model architecture for image segmentation tasks is U-Net (14), as it consists of several convolutional layers in both downsampling and upsampling paths while using skip connections between the layers in those paths to maintain high resolution and fine details of the model's inputs throughout its training time (9). U-Net's decoder can produce attention maps or recreate the input images depending on how the model is trained, and hence U-Net architecture is often desirable for joint image classification and localization tasks. Other architectures that can be used for image localization include YOLO, mask R-CNN, and faster R-CNN (9).

To tackle the localization problem in the case where the annotated datasets are not large, the use of the combinations of different techniques, such as a classifier and a localizer that produces bounding boxes and probability heatmaps, is shown to be effective (26). Some notable methods include Weakly Supervised Learning (27–29), Self-Transfer Learning (30), and other models, such as those introduced in (31).

For example, a proposed weakly supervised learning method in (27) consists of a three-stage network for disease localization that first generates class activation maps, and then feeds those maps into a network that outputs pseudo labels. Finally, the method uses the generated pseudo labels for image segmentation.

As another example, a self-transfer learning framework introduced in (30) consists of three components (convolutional layers, fully connected classification layers, and localization layers), and its goal is to perform image localization. The classification branch and localization branch of the model are trained simultaneously with two losses. To ensure that the localization component does not stray away, the weights on the classification loss decrease through the training process while the weights on the localization loss increase.

Although saliency maps are often produced and analyzed post-training, there are several architectures that incorporate attention maps and map-generating mechanisms in model training to improve explainaibility and classification or segmentation performance, such as those introduced in (32–36).

Kazemimoghadam et al. (32) incorporated saliency maps as one of the inputs to multiple U-Net models for post-operative tumor bed volume segmentation in CT images for breast radiotherapy. Specifically, the saliency maps are encoded using markers in CT images, which will be combined with the CT images to guide the model to focus on higher intensity values in the saliency maps to extract relevant features for a more accurate segmentation. Ultimately, voting occurs among multiple U-Net models to produce a final segmentation prediction.

Unlike how (32) focuses on using saliency maps for a specific medical use case, (33) focuses on the general perspective on attention maps and addresses that the existing saliency map generators result in noisy maps. Since the model gradients should highlight mostly only the relevant features for consistent and accurate performance, (33) proposed saliency-guided training that diminishes gradients on irrelevant features without worsening model performance. This is done through masking input features that results in lower gradient values, resulting in more sparse, precise gradients. (33) also provides experiments that incorporate the saliency-guided training on various modals (image, language, and time series) for classification tasks to show the framework's effectiveness.

Wang et al. (35), on the other hand, recognizes that there is substantial amount of overlaps between class-specific attention maps, which could lead to more “visual confusion” for models and classification errors. Hence (35), establishes an end-to-end pipeline (called ICASC, Improving Classification with Attention Separation and Consistency) that provides the discrimination of class-specific attention and enforces the discriminative features to be consistent across models' CNN layers to improve the overall classification performance.

Li et al. (34) also developed an end-to-end network (called GAIN, Guided Attention Inference Network), which supervises a model's attention maps during training to guide the model to make predictions based on relevant features of the inputs. (34) states that GAIN was developed to tackle the problem that attention maps (that are generated using only classification labels), which are used as priors for segmentation or localization tasks, tend to only cover smaller regions, and hence the maps can be incomplete and less accurate. GAIN uses two network streams, one is for classification and the other is for attention mining, where the classification stream helps the other by providing information on areas of the inputs associated to classification task, while the other ensures that all relevant parts are incorporated during classification.

Overall, there is no one single architecture that outperforms others in terms of multi-task classification and segmentation especially for medical domain, but prior work shows that ensemble learning or multi-stage training performs better in general for the multi-tasking (9). Additionally, much of the published works incorporate transfer learning done on large image datasets that contain common objects or organisms, but they are not much work done on creating transferrable models trained on medical datasets (30). To the best of our knowledge, this research is the first to directly use GBP and deconvNet heatmap generators and eye-gaze information in model training to guide the U-Net model to improve disease classification performance and to produce attention maps similar to how the radiologists view CXR images for diagnosis.

## Dataset and methods

This section first gives an overview of the dataset (12) used for this study and introduces two baseline experiments that are established in (12), which showcased the usability of the dataset. One of the baseline experiments and its corresponding results are treated as the baseline model and baseline results for this study. The comparison and analysis of the baseline experiments’ results are also detailed. Secondly, this section further illustrates the motivation behind this research, which came from assessing the effectiveness of adding a segmentation component to a classifier. Finally, this section outlines this study's three proposed models, their architectures, and their training methods.

### Dataset and baseline experiments

#### Dataset

**Figure 1:**
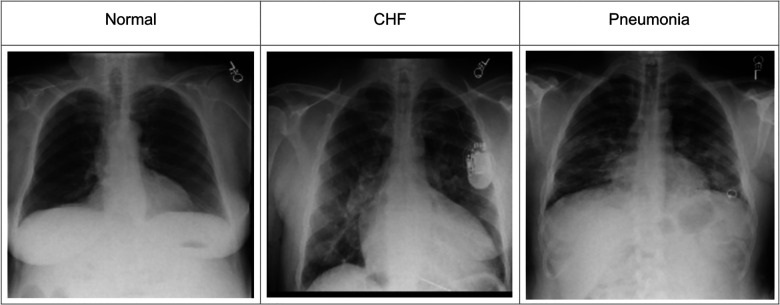
Example CXR images for each of the three classes from (12).

To incorporate the heatmap generators and radiologist's eye-gaze data on CXR images when training deep learning models, this work used a dataset that contains 1,083 chest radiographs, which preserve the high image quality as DICOM files. The images were reviewed and reported by one radiologist (12). The dataset also contains the transcribed radiology report, the radiologist's dictation audio and eye gaze coordinates mapped onto the corresponding images, and the associated disease class labels. The class labels include “normal”, “CHF”, and “pneumonia”. Several CXR image examples of each of the classes in the dataset can be seen in Figure 1. The labels were all from formal clinical diagnoses, and the dataset contains an equal number of datapoints for each class. Because there was a misalignment between the csv files provided in the dataset and several image IDs were missing when generating eye-gaze heatmaps, all the experiments ran in this work dealt with 1,017 images from the dataset and their corresponding eye-gaze information. A list of the 1,017 images used in this study is available on our Github repository^1^. For training, this dataset was split into training, validation, and test sets with the percentage of 80, 10, and 10, respectively. When splitting the data, unique patient IDs were in only one of the training, validation, or test datasets to prevent potential biases.

**Figure 2:**
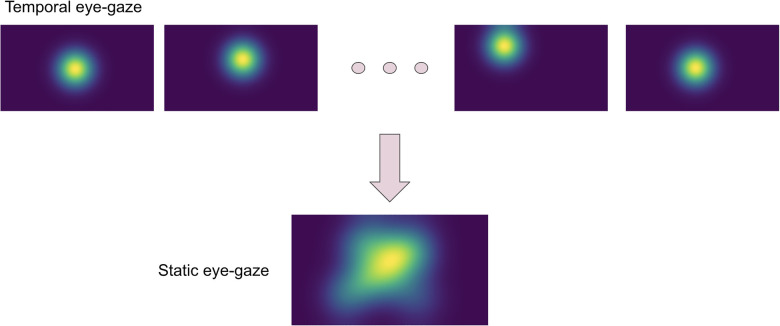
Temporal and static eye-gaze heatmaps.

The eye-tracking data was used to teach the model about how radiologists observed the CXR images. Thus, the eye-gaze coordinates and fixation data were utilized to generate temporal and static eye-gaze heatmaps (as can be seen in Figure 2) using an open-sourced code (12) where static heatmaps are concatenations of the corresponding temporal heatmaps.

#### Baseline experiments

The paper (12) that introduced the dataset included two experiments that were conducted to show how the dataset's eye-gaze information could be used when training a deep learning model for a disease classification task. The models' performance was assessed using the area under the receiver operating characteristic curve (AUC).

**Figure 3:**
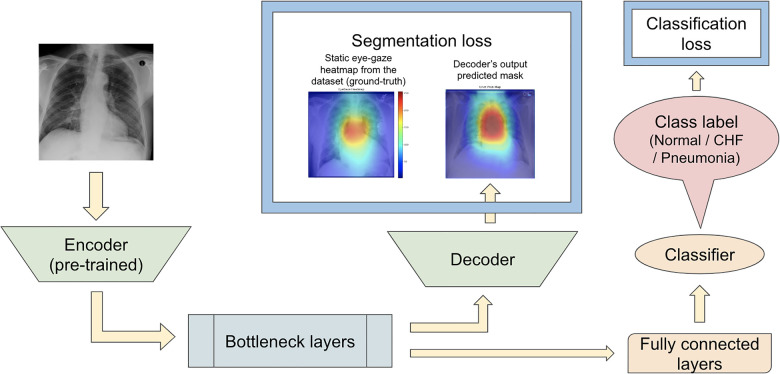
(12)’s second experiment's U-Net model architecture with the radiologist's static eye-gaze data used as the ground truths for the decoder's outputs when computing the segmentation loss.

Karargyris et al. (12) used both the DICOM CXR images and the temporal eye-gaze heatmaps as part of the inputs to the model to operate disease classification. This experiment was created to show how the temporal eye-gaze data in addition to the chest radiographs can be fed into a deep learning model as inputs. Their second set of experiments was created to show how the eye-gaze data can be used for explainability purposes by using them in training so that the model's decoder can produce probability maps that look similar to the static eye-gaze heatmaps. It consisted of a U-Net structure as shown in Figure 3, with convolutional encoder and bottleneck layers that use pre-trained EfficientNet-b0 (37, 38), a classification head, and a convolutional decoder. It computed and combined two sets of losses (both using Binary Cross Entropy with Logits Loss function); one was classification loss from the classification head, and the other was a segmentation loss computed between the static eye-gaze heatmap of the corresponding CXR image and the U-Net's output predicted heatmap from the decoder. The average AUC values achieved using this method are shown in “U-Net (treated as the baseline model for this research)” section of Table 1. It appears that the model was able to classify the “normal” condition the best, while it struggled most to correctly classify the “pneumonia” condition.

**Table 1:** Comparing the AUC values between (12)'s baseline model with no segmentation component and the U-Net architecture (the value preceding the parenthesis is the 50th percentile value and the values in parenthesis are 2.5th and 97.5th percentile values, and the value after a semicolon is the *p*-value).

Model Details	Average AUC	“Normal” AUC	“CHF” AUC	“Pneumonia” AUC
U-Net (treated as the baseline model for this research)	0.872 (0.840, 0.897)	0.923 (0.895, 0.945)	0.916 (0.871, 0.938)	0.781 (0.713, 0.851)
Model without the segmentation component	0.873 (0.838, 0.908); 0.128	0.878 (0.836, 0.918); 7.068 × 10^−14^	0.934 (0.898, 0.975); 4.774 × 10^−3^	0.805 (0.763, 0.886); 0.273

**Figure 4:**
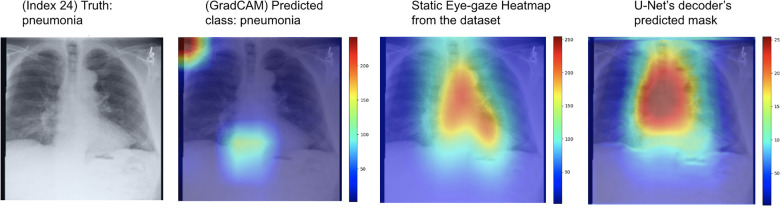
Heatmaps generated from Grad-CAM and the (12)'s U-Net's decoder for a pneumonia class example correctly classified.

One example of the generated heatmaps using the trained U-Net model from (12) given a CXR image can be seen in Figure 4. In this paper, the terms generated heatmap and predicted heatmap refer to the output of a U-Net decoder which is trained for generating heatmaps. Additionally, derived or calculated heatmap refers to the results of GBP or deconvNet applied to a model. The leftmost image is the model's input chest radiograph; the inner left heatmap is the output of running Grad-CAM after training; the inner right heatmap is the static eye-gaze heatmap from the dataset that was used as the ground truth when computing the segmentation loss; and the rightmost heatmap is the model's predicted probability mask generated from the U-Net's decoder. Although the U-Net's predicted heatmaps seemed to be trained well to align more with the static eye-gaze heatmaps as was expected from how the segmentation loss was computed, the Grad-CAM results for explainability purposes did not visually overlap well with the ground truth static eye-gaze heatmaps, and hence the model's focus when making classification predictions seemed to not necessarily be similar to the radiologist's eye-gaze focus when making diagnosis. This U-Net architecture, which used static eye-gaze heatmaps as part of the segmentation loss computation, and its corresponding AUC values were treated as the baseline model and baseline AUC values for this study.

#### Motivation for this study

**Figure 5:**
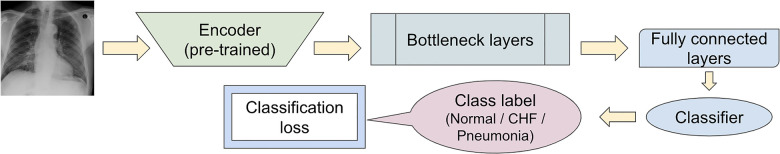
Model architecture of the baseline model provided in (12) for the static eye-gaze experiment without the segmentation component.

To observe if adding the segmentation component to the disease classifier contributes to improving the overall classification AUC values, the performances of the U-Net architecture (Figure 3) and a model without the segmentation component (Figure 5) were compared using the classification AUC values. The model without the segmentation component was the baseline model that (12) provided for the static eye-gaze heatmap experiment. This model did not attempt to enhance the explainability component simultaneously and only focused on the classification task. The classification performance of the non-segmentation model and the U-Net using the AUC metric, which is depicted in Table 1, shows that the overall average AUC values are similar for both models. From these AUC results, it appears that the multitasking of improving classification and incorporating the explainability component to model training does not result in improved mean AUC values. Nevertheless, when the AUC values for each of the three classes were viewed separately, it was concluded that U-Net architecture improved to classify “normal” condition better than the non-segmentation model, but “CHF” and “pneumonia” classes had lower AUC values compared to those of the non-segmentation model. Such observations suggest that there could be improvements made for this U-Net architecture that would incorporate the explainability component when attempting to improve the overall classification AUC, particularly for the “CHF” and “pneumonia” classes.

### Defining segmentation loss using heatmap generators

Given (12)'s two experiments, the dataset, and the different heatmap generators for explainability improvement, this study produced and experimented with three sets of proposed models using the (12)'s U-Net architecture to observe if using both the eye-gaze data and various heatmap generators during model's training time could result in improved AUC for the classification task while enhancing the predicted heatmap generation. Specifically, the experiments involved using the heatmap generators (such as GBP and deconvNet) during training and different ways of using the calculated heatmaps when computing the segmentation loss. All the proposed model sets used Binary Cross Entropy with Logits Loss function when computing the segmentation loss as how (12) used it.

Since the major goal was to improve the classification AUC, the average AUC value and the AUC values for each of the three classes were used to evaluate the models' performance on each of the experiments. Finally, the 95% confidence interval (CI) (with 2.5th % and 97.5th % values) in addition to the average AUC values were measured for each of the three class labels over 55–60 samples with resampling for 30 iterations using the test set.

#### Proposed model set 1

**Figure 6:**
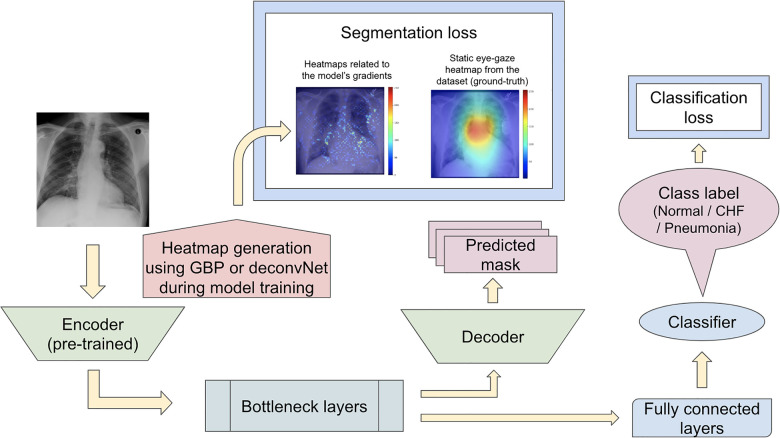
Model architecture for the proposed model set 1 with the segmentation loss computed based on derived heatmaps using either GBP or deconvNet and the static eye-gaze ground truth heatmaps from the dataset.

For the first proposed model set, the segmentation loss was computed using heatmaps generated from the different generators (which included GBP and deconvNet) during the training time against the static eye-gaze heatmaps obtained from the dataset, which were treated as ground truth, as can be seen in Figure 6. This experiment attempted to guide the model's trainable parameters to mimic the static eye-gaze heatmaps so that the model could learn to focus on similar areas of the chest radiographs as how the radiologist did.

#### Proposed model set 2

**Figure 7:**
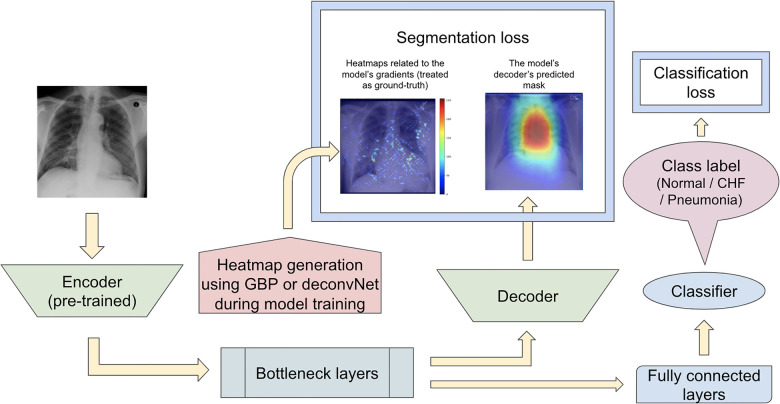
Model architecture for the proposed model set 2 with the segmentation loss computed based on derived heatmaps using either GBP or deconvNet and the outputs from the U-Net's decoder.

The second proposed model set was run with another modification to the network training when computing the segmentation loss. In contrast to the previous proposed model that computed the segmentation loss using the heatmaps generated from either GBP or deconvNet and the static eye-gaze heatmaps, the segmentation loss for this proposed model was computed using the differences between the outputs of the U-Net's decoder and heatmaps generated from either GBP or deconvNets, as can be seen in Figure 7. This experiment was run to assess if the model's decoder can be trained to generate heatmaps consistent with the outputs from either GBP or decondNet.

#### Proposed model set 3

**Figure 8:**
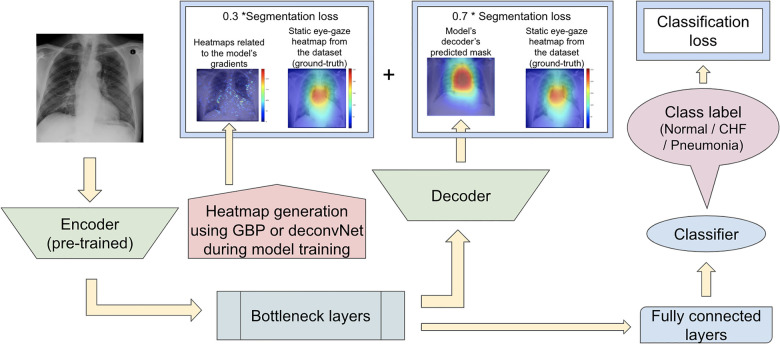
Model architecture for the proposed model set 3 with the segmentation loss computed using the weighted average of the two loss coponents.

Since the previous two proposed model sets did not focus on training the model's decoder to generate predicted heatmaps that appear similar to the gound-truth static eye-gaze heatmaps, the third proposed model set used a weighted average of two segmentation losses, where one of which was computed using the differences between the U-Net's generated masks and the dataset's static eye-gaze heatmaps, while the other loss was computed using the differences between the heatmaps derived based on GBP or deconvNet and the dataset's static eye-gaze heatmaps as shown in Figure 8. This modification in the segmentation loss computation incorporated the different heatmap generators so that the model's trainable parameters would learn to focus on similar areas as the ground truth static eye-gaze heatmaps, while simultaneously guiding the model's decoder to generate predicted heatmaps that look similar to the ground truth static eye-gaze heatmaps. The ratio of the combination of the two segmentation losses was treated as an adjustable hyperparameter. This method of computing the segmentation loss emphasizes how the radiologist viewed the CXR images during model training.

## Results

### Proposed model set 1

For the first experiment set, the use of GBP as the heatmap generator when computing the segmentation loss resulted in the highest average AUC values compared to the AUC values obtained using deconvNet and the baseline AUC values as can be seen in Table 2. This difference between the use of GBP and deconvNets is understandable due to the nature of the computation differences between GBP and deconvNets, where GBP is based on deconvNet but sets the negative gradients for inputs to zero, and hence GBP highlights the important regions of inputs even more (39). For both GBP and deconvNet cases, the average AUC values were higher than that of the baseline model, and the greatest improvement in AUC values occurred for classifying “pneumonia” with 5% increase when using GBP and 2.5% increase when using deconvNet. Additionally, classifying “CHF” also had 3% improvement when using GBP and 1.9% improvement when using deconvNet. Although both “CHF” and “pneumonia” classification improved in this experiment set with the use of the heatmap generators during the model's training time, this experiment did not result in a great improvement for average AUC values because the AUC values for classifying the “normal” class was lower with the use of the heatmap generators when computing segmentation loss compared to the baseline values.

**Table 2:** Comparing the output AUC values between (12)'s U-Net model and a U-Net with different derived heatmaps (GBP or deconvNet) for the Experiment Set 1 (the value preceding the parenthesis is the 50th percentile value and the values in parenthesis are 2.5th and 97.5th percentile values, and the value after a semicolon is the *p*-value).

Model and heatmap generator's details	Average AUC	“Normal” AUC	“CHF” AUC	“Pneumonia” AUC
U-Net (baseline)	0.872 (0.840, 0.897)	0.923 (0.895, 0.945)	0.916 (0.871, 0.938)	0.781 (0.713, 0.851)
Guided back-propagation	0.891 (0.847, 0.939); 3.210 × 10^−2^	0.896 (0.854, 0.945); 1.086 × 10^−3^	0.946 (0.891, 0.979); 1.417 × 10^−4^	0.831 (0.763, 0.923); 4.471 × 10^−4^
DeconvNet	0.884 (0.843, 0.935); 0.874	0.916 (0.884, 0.943); 8.061 × 10^−6^	0.935 (0.905, 0.959); 8.855 × 10^−4^	0.806 (0.715, 0.918); 0.707

**Figure 9:**
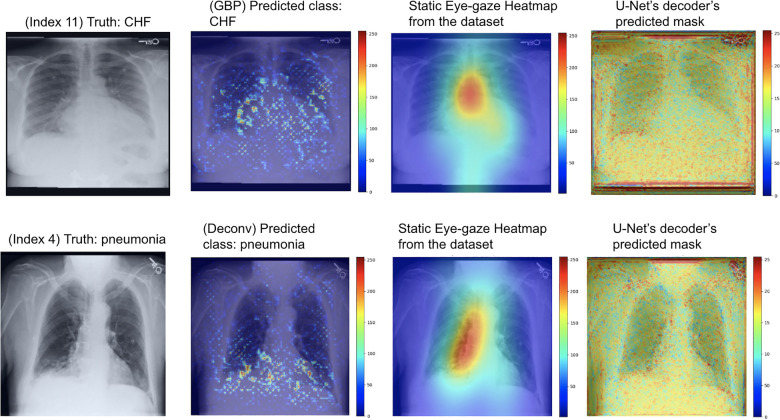
Model's output heatmaps using GBP (top) and deconvNet (bottom) as the heatmap generator for Experiment Set 1 for the correctly classified CHF (top) and pneumonia (bottom) classes.

As can be seen in Figure 9, the GBP (shown in the top row) and deconvNet's (shown in the bottom row) heatmaps highlighted areas near the lungs and heart similarly to the ground truth static eye-gaze heatmaps. Additionally, there was a correlation between the greater vividness of GBP's heatmaps and the slightly higher AUC values compared to those from deconvNets as can be seen in Table 2. However, the model's predicted heatmaps appeared to be quite different from that of static eye-gaze heatmaps. This was because the segmentation loss was computed without the U-Net's predicted heatmap in attempts to align the model's parameters to highlight similar areas as the static eye-gaze heatmaps, and hence the decoder was not trained to produce predicted heatmaps that appear similar to the static eye-gaze heatmaps.

### Proposed model set 2

Similarly to the first proposed model set, the GBP method performed the best compared to the baseline and the deconvNet method for the proposed model set 2. Furthermore, the outcome of using GBP for this had a slight improvement in the average AUC value compared to the first one. The greatest improvement occurred for the “pneumonia” class AUC, with 6.9% increase in AUC, which contributed to the 2.5% increase in the average AUC values when using GBP compared to the baseline AUC values.

**Figure 10:**
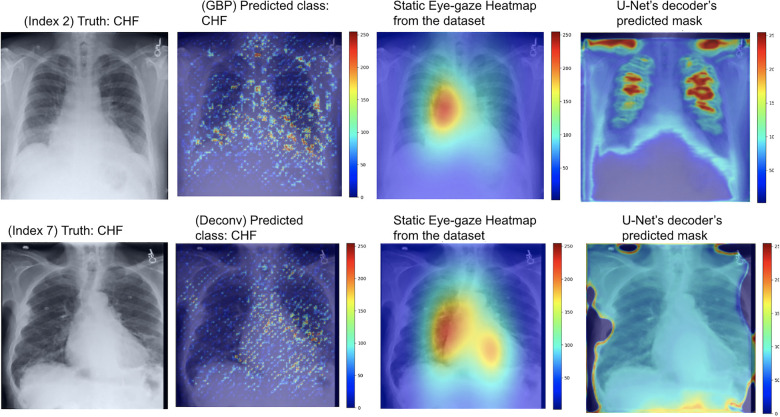
Model's output heatmaps using GBP (top) and deconvNet (bottom) as the heatmap generator for Experiment Set 2 for the correctly classified CHF class.

For this experiment, the U-Net was trained so that the decoder would attempt to mimic the heatmaps derived from the GBP or deconvNet when outputting its predicted heatmap, which can be observed in the rightmost images Figure 10. The use of GBP as the heatmap generator (outputs shown in the top row of Figure 10) resulted in the U-Net's generated masks having higher intensity than those produced when using deconvNets (outputs shown in the bottom row of Figure 10).

Although the U-Net's predicted heatmaps appeared to not mimic the heatmaps derived using GBP or deconvNet, there seems to be a correlation among the heatmaps' intensity differences and the differences in the U-Net's predicted heatmaps' intensity and in the locations of focus when using different heatmap generators. Additionally, there was a correlation between the greater intensity of the calculated heatmaps from GBP or deconvNet and the predicted heatmaps and the higher average AUC values when comparing the use of GBP and deconvNet, which can be seen in Table 3.

**Table 3:** Comparing the output AUC values between (12)'s U-Net model and a U-Net with different derived heatmaps (GBP or deconvNet) for the Experiment Set 2 (the value preceding the parenthesis is the 50th percentile value and the values in parenthesis are 2.5th and 97.5th percentile values, and the value after a semicolon is the *p*-value).

Model and heatmap generator's details	Average AUC	“Normal” AUC	“CHF” AUC	“Pneumonia” AUC
U-Net (baseline)	0.872 (0.840, 0.897)	0.923 (0.895, 0.945)	0.916 (0.871, 0.938)	0.781 (0.713, 0.851)
Guided back-propagation	0.897 (0.848, 0.921); 1.410 × 10^−3^	0.907 (0.863, 0.946); 3.732 × 10^−2^	0.922 (0.888, 0.956); 0.103	0.850 (0.792, 0.900); 8.989 × 10^−9^
DeconvNet	0.881 (0.828, 0.907); 0.776	0.885 (0.836, 0.928); 2.932 × 10^−11^	0.922 (0.890, 0.952); 0.412	0.827 (0.732, 0.901); 6.953 × 10^−3^

### Proposed model set 3

This experiment with the combination of two segmentation losses incorporated the heatmap generators in training time to guide the model's parameters to focus on similar areas as how the radiologist viewed, while guiding the model to produce predicted heatmaps that look similar to the static eye-gaze heatmaps for explainability purposes.

**Figure 11:**
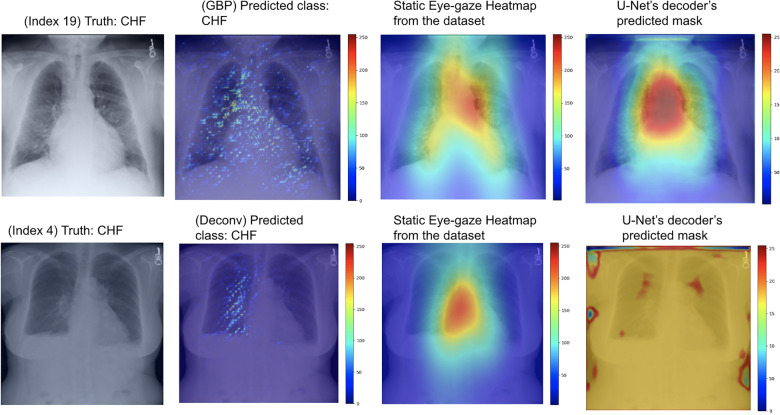
Model's output heatmaps using GBP (top) and deconvNet (bottom) as the heatmap generator for Experiment Set 3 for the correctly classified CHF class.

As can be seen in Table 4, the use of deconvNet resulted in lower AUC values than the baseline model except for the “CHF” classification, which improved by 1.8%. These AUC results appear to be associated with how the U-Net's decoder was unable to mimic the static eye-gaze heatmaps when creating the predicted heatmap, as can be seen in the bottom row of Figure 11.

**Table 4:** Comparing the output AUC values between (12)'s U-Net model and a U-Net with different derived heatmaps (GBP or deconvNet) for the Experiment Set 3 (the value preceding the parenthesis is the 50th percentile value and the values in parenthesis are 2.5th and 97.5th percentile values, and the value after a semicolon is the *p*-value).

Model and heatmap generator's details	Average AUC	“Normal” AUC	“CHF” AUC	“Pneumonia” AUC
U-Net (baseline)	0.872 (0.840, 0.897)	0.923 (0.895, 0.945)	0.916 (0.871, 0.938)	0.781 (0.713, 0.851)
Guided back-propagation	0.913 (0.860, 0.966); 1.281 × 10^−6^	0.921 (0.866, 0.968); 1.981 × 10^−16^	0.962 (0.933, 0.989); 0.684	0.859 (0.732, 0.957); 5.144 × 10^−79^
DeconvNet	0.860 (0.806, 0.907); 0.213	0.907 (0.856, 0.962); 3.928 × 10^−4^	0.934 (0.891, 0.974); 5.039 × 10^−7^	0.741, (0.643, 0.840); 5.239 × 10^−2^

On the contrary, the use of GBP for this segmentation loss computation had improvements in most of the AUC values, and the model's decoder was also able to generate the predicted heatmap that highlighted similar areas as the static eye-gaze heatmaps as shown in the top row of Figure 11. Specifically, when using GBP, the average AUC value improved by 4.1%, the “CHF” classification improved by 4.6%, and the “pneumonia” classification improved by 7.8% compared to the baseline model that did not use the heatmap generators during the training time. Hence, this modification in the segmentation loss computation using GBP produced the best AUC values across all of the experiments in this research.

Overall, the vividness of the heatmaps derived using GBP or deconvNet and the similarity between the U-Net's predicted heatmaps and the dataset's static eye-gaze heatmaps seemed to be correlated with how well the average AUC values improved for the experiments. This further confirms that the radiologist's eye-gaze information is valuable in improving the classification performance when used with the heatmap generators during model training for multitasking.

## Discussion

AI models for radiology are improving dramatically for disease classification or localization tasks, but there is often a trade-off between performance and explainability, as higher performing models tend to be deeper and more complex. Hence, explainability for AI in medical domain is gaining more attention to give insight into how the models arrived at their predictions and to increase trust in the use of such models. Additionally, from the legal point of view, although explainability is currently not a strict requirement for the AI use in clinical situations, food and drug administration states that some level of transparency, which can include open communication regarding inputs and outputs of the AI model and its algorithm, is required to ensure transparency to the patients and the physicians (6). Furthermore, explainaibility will likely become a stricter requirement as more AI models are incorporated into clinical use (40). Therefore, this study highlights integrating the explainability aspect into both model training and model outputs with the goal of improving disease classification.

There exist several AI studies that together classify diseases and generate heatmaps. Nonetheless, they have several issues. For example, the publicly available datasets that such research uses sometimes contain incorrect labels obtained from NLP, and the research oftentimes use only the CXR images and class labels without incorporating the other methods or data the radiologists usually use when making diagnoses.

Thus, this research proposed three model sets that take a U-Net architecture with distinct loss function computations using a dataset that contains eye-gaze information from a radiologist. This study showed that using a radiologist's eye-gaze data and derived heatmap (specifically GBP or deconvNet) in model training can improve disease classification AUC, especially for “CHF” and “pneumonia” classes that help reduce false negatives. Moreover, by guiding the model's gradients to align to the radiologist's eye-gaze heatmaps, the model is able to produce enhanced heatmaps that can be used for explainability purposes. Furthermore, this study confirms that using different types of data other than just the CXR images and disease class labels and following radiologists' methods for diagnosis when establishing and training AI models can boost the classification performance.

The heatmap visualization outputs of the three proposed model sets highlight areas of CXR images that the model focused on when making predictions. The outputs of the first proposed model set for both GBP and deconvNet show that each generator appeared to focus on similar areas as the static eye-gaze heatmaps did, but the U-Net's predicted heatmaps seemed to not focus well, which is understandable due to the way the segmentation loss was calculated for this model set.

The outputs of the second proposed model set using deconvNet as the heatmap generator (shown in the bottom row of Figure 10) convey that since the derived heatmap showed more sparse intensities compared to the dataset's static eye-gaze heatmaps, the intensity of the model's predicted heatmaps was also lower and covered a wider area of the chest compared to the outputs from the other experiments. On the contrary, there seemed to be a correlation between the greater vividness and intensity for U-Net's predicted heatmap and the higher AUC values when comparing between the use of GBP or deconvNet.

Finally, the visualization outputs of the third proposed model set clearly showcased that the use of GBP method as the heatmap generator for this training method performed superior compared to others. Both the U-Net's predicted heatmaps and the heatmaps created from GBP focused on similar areas as the eye-gaze heatmaps, suggesting that the model is learning to produce explainable heatmaps while predicting the disease class.

Viewing the results from the three experiment sets, particularly focusing on the AUC values in Table 4, the multitasking of improving classification and generating reasonable heatmaps for enhancing explainability during model training using the U-Net architecture was shown to be effective when applying GBP as the heatmap generator. Although the confidence intervals of the AUC values for the proposed model set 3's GBP method had some overlap with the baseline's confidence intervals, this new method not only had improvements in the average AUC value (4.1%), but it also had greater improvements for the “CHF” (4.6%) and “penumonia” (7.8%) classes, which were the classes the baseline struggled to classify. Since the major focus for disease classification tasks is on correctly identifying the non-“normal” disease classes and decreasing the false negatives, these improvements with the use of GBP heatmap generator during the training time was significant. Furthermore, the experiments' results suggest that the use of heatmap generators (particularly GBP) in training time could also enhance the model's predicted heatmaps generation for explainability purposes to better convey where the model's attention was on the input chest radiographs.

The major limitation of this research is the small size of the dataset and the potential bias the dataset may have because the eye-gaze data was collected from, and the diagnosis was done by one radiologist (12). Thus, other medical imaging datasets that are larger and contain eye-gaze information from multiple radiologists may be used to further validate the disease classification improvements produced by using heatmap generators and eye-gaze data during model training. Furthermore, with other datasets, the out-of-distribution generalization of the model should be confirmed in the future studies to assess the model's potential biases (41).

To further validate the classification decisions this study's models made and to increase trust in the model's predictions, DOCTOR's Totally Black Box scenario (42) can be used after the model makes class predictions on given CXR images. DOCTOR is a discriminator that can be used to detect if each of the predictions made by the AI model can be trusted or not irrespective of in- or out- distribution the data is coming from, and it does not require any prior knowledge on the dataset or model architecture. DOCTOR's decisions can signal physicians to take a second look at the CXR images and the associated class prediction the model made if DOCTOR rejects the predictions. When applying DOCTOR, there will be a trade-off between the number of samples that are rejected and the threshold value for false detection and acceptance rate, and ensuring that more misclassified samples are being rejected results in more samples, including some that are correctly classified, being rejected as well. Nonetheless, employing DOCTOR in this research's model will be valuable because it is essential to eliminate model's misclassifications particularly in radiology field and the overall medical domain.

## Conclusion

In this research, three proposed model sets that use heatmap generators in the U-Net model's training time were investigated for the radiology field to simultaneously improve the disease classification performance and better highlight the model's attention spots by generating explainable heatmaps. The experiments used a dataset that contain chest radiographs, eye-gaze coordinates from one radiologist, and the corresponding images' class labels. The confidence intervals of the AUC values for each experiment that incorporated the static eye-gaze information and the heatmap generators during the model's training time were computed. The proposed model in Figure 8 that used the weighted average of two segmentation losses (one computed between the heatmaps generated from GBP and the dataset's static eye-gaze heatmaps, and the other computed between the predicted heatmaps from the U-Net's decoder and the static eye-gaze heatmaps) performed superior compared to the baseline model provided in (12) and the other models that were tested in this research. As a future work, a larger medical imaging dataset with eye-gaze information from multiple radiologists could be used to further validate the disease classification improvement achieved by this method. Moreover, DOCTOR can be employed to estimate how much each of the predictions made by the model is trustworthy or not to further increase trust in the model's predictions.

## Data Availability

Publicly available datasets were analyzed in this study. This data can be found here: https://physionet.org/content/egd-cxr/1.0.0/.

## Footnotes

^1^ https://github.com/IMICSLab/Classification_and_Explainability_in_Radiology

## References

[1] Linardatos P Papastefanopoulos V Kotsiantis S. Explainable AI: a review of machine learning interpretability methods. Entropy. (2021) 23(1):18. 10.3390/e23010018 PMC7824368 33375658

[2] Singh A Sengupta S Lakshminarayanan V. Explainable deep learning models in medical image analysis. J Imaging. (2020) 6(6):52. 10.3390/jimaging6060052 34460598 PMC8321083

[3] Angelov P Soares E Jiang R Arnold N Atkinson P. Explainable artificial intelligence: an analytical review. Wiley Interdiscip Rev. (2021) 11(5):e1424. 10.1002/widm.1424

[4] Fuhrman JD Gorre N Hu Q Li H Naqa I Giger ML. A review of explainable and interpretable AI with applications in COVID-19 imaging. Med. Phys. (2022) 49:1–14. 10.1002/mp.15359 34796530 PMC8646613

[5] Holzinger A Biemann C Pattichis CS Kell DB. What do we need to build explainable AI systems for the medical domain? arXiv preprint arXiv:1712.09923.

[6] Amann J Blasimme A Vayena E Frey D Madai V. Explainability for artificial intelligence in healthcare: a multidisciplinary perspective. BMC Med Inform Decis Mak. (2020) 310. 10.1186/s12911-020-01332-6 PMC7706019 33256715

[7] Reyes M Meier R Pereira S Silva CA Dahlweid F von Tengg-Koblig H On the interpretability of artificial intelligence in radiology: challenges and opportunities. Radiol Artif Intell. (2020) 2(3). 10.1148/ryai.2020190043 32510054 PMC7259808

[8] Fernandez-Quilez A. Deep learning in radiology: ethics of data and on the value of algorithm transparency, interpretability and explainability. AI Ethics. (2022) 2. 10.1007/s43681-022-00161-9

[9] Çallı E Sogancioglu E van Ginneken B van Leeuwen KG Murphy K. Deep learning for chest x-ray analysis: a survey. Med Image Anal. (2021) 72:102125. 10.1016/j.media.2021.102125 34171622

[10] Shad R Cunningham J Ashley EA Langlotz CP Hiesinger W. Medical imaging and machine learning. arXiv:2103.01938.

[11] Kusakunniran W Karnjanapreechakorn S Siriapisith T Borwarnginn P Sutassananon K Tongdee T COVID-19 detection and heatmap generation in chest x-ray images. J Med Imaging. (2021) 8:014001. 10.1117/1.JMI.8.S1.014001 PMC7804292 33457446

[12] Karargyris A Kashyap S Lourentzou I Wu JT Sharma A Tong M Creation and validation of a chest x-ray dataset with eye-tracking and report dictation for AI development. Sci Data. (2021) 8(1):92. 10.1038/s41597-021-00863-5 33767191 PMC7994908

[13] Rong Y Xu W Akata Z Kasneci E. Human Attention in Fine-grained Classification. arXiv:2111.01628. (2021).

[14] Ronneberger O Fischer P Brox T. U-net: convolutional networks for biomedical image segmentation. In: Navab N Hornegger J Wells W Frangi A, editors. Medical image computing and computer-assisted intervention—MICCAI 2015. Cham: Springer (2015). p. 234–41.

[15] Preechakul K Sriswasdi S Kijsirikul B Chuangsuwanich E. Improved image classification explainability with high-accuracy heatmaps. iScience. (2022) 25(3):103933. 10.1016/j.isci.2022.103933 35252819 PMC8889368

[16] Salahuddin Z Woodruff HC Chatterjee A Lambin P. Transparency of deep neural networks for medical image analysis: a review of interpretability methods. Comput Biol Med. (2022) 140:105111. 10.1016/j.compbiomed.2021.105111 34891095

[17] Selvaraju RR Cogswell M Das A Vedantam R Parikh D Batra D. Grad-CAM: visual explanations from deep networks via gradient-based localization. 2017 IEEE international conference on computer vision (ICCV) (2017). p. 618–26. 10.1109/ICCV.2017.74

[18] Zeiler MD Fergus R. Visualizing and understanding convolutional networks. In: Fleet D Pajdla T Schiele B Tuytelaars T, editors. Computer vision—ECCV 2014. Cham: Springer (2014). p. 818–33.

[19] Springenberg JT Dosovitskiy A Brox T Riedmiller MA. Striving for Simplicity: The All Convolutional Net. CoRR. abs/1412.6806. arXiv:1412.6806 (2015).

[20] Zhou B Khosla A Lapedriza A Oliva A Torralba A. Learning deep features for discriminative localization. 2016 IEEE conference on computer vision and pattern recognition (CVPR) (2016). p. 2921–9, 10.1109/CVPR.2016.Z319

[21] Mohamed E Sirlantzis K Howells G. A review of visualization-as-explanation techniques for convolutional neural networks and their evaluation. Displays. (2022) 73:102239. 10.1016/j.displa.2022.102239

[22] Chattopadhay A Sarkar A Howlader P Balasubramanian VN. Grad-CAM++: generalized gradient-based visual explanations for deep convolutional networks. 2018 IEEE winter conference on applications of computer vision (WACV) (2018). p. 839–47, 10.1109/WACV.2018.00097

[23] Smilkov D Thorat N Kim B Viegas F Wattenberg M. Smoothgrad: removing noise by adding noise. arXiv:1706.03825 (2017).

[24] Shrikumar A Greenside P Kundaje A. Learning important features through propagating activation differences. Proceedings of the 34th international conference on machine learning—volume 70 (ICML’17). JMLR.org. (2017). p. 3145–53.

[25] Ribeiro MT Singh S Guestrin C. “Why should I trust you?”: explaining the predictions of any classifier. Proceedings of the 22nd ACM SIGKDD international conference on knowledge discovery and data mining (KDD ‘16). New York, NY, USA: Association for Computing Machinery (2016). p. 1135–44. 10.1145/2939672.2939778

[26] Pesce E Withey SJ Ypsilantis PP Bakewell R Goh V Montana G. Learning to detect chest radiographs containing pulmonary lesions using visual attention networks. Med Image Anal. (2019) 53:26–38. 10.1016/j.media.2018.12.007 30660946

[27] Viniavskyi O Dobko M Dobosevych O. Weakly-supervised segmentation for disease localization in chest x-ray images. Artificial intelligence in medicine: 18th international conference on artificial intelligence in medicine, AIME 2020, Minneapolis, MN, USA (2020). Proceedings. Springer-Verlag, Berlin, Heidelberg, p. 249–59. 10.1007/978-3-030-59137-3_23

[28] Chaudhry A Dokania PK Torr P. Discovering class-specific pixels for weakly-supervised semantic segmentation. Procedings of the British machine vision conference 2017 (2017). 10.5244/c.31.20

[29] Wei Y Feng J Liang X Cheng MM Zhao Y Yan S. Object region mining with adversarial erasing: a simple classification to semantic segmentation approach. 2017 IEEE conference on computer vision and pattern recognition (CVPR) (2017). 10.1109/cvpr.2017.687

[30] Hwang S Kim HE. Self-transfer learning for weakly supervised lesion localization. In: Ourselin S Joskowicz L Sabuncu M Unal G Wells W, editors. Medical image computing and computer-assisted intervention—mICCAI 2016. Cham: Springer (2016) 239–46. 10.1007/978-3-319-46723-8_28

[31] Rajpurkar P, Irvin J, Zhu K, Yang B, Mehta H, Duan T

[32] Kazemimoghadam M Chi W Rahimi A Kim N Alluri P Nwachukwu C Saliency-guided deep learning network for automatic tumor bed volume delineation in post-operative breast irradiation. Phys Med Biol. (2021) 66(17):10.1088/1361-6560/ac176d. 10.1088/1361-6560/ac176d PMC8639319 34298539

[33] Ismail AA Bravo HC Feizi S. Improving Deep Learning Interpretability by Saliency Guided Training. arXiv:2111.14338.

[34] Li K Wu Z Peng KC Ernst J Fu Y. Tell me where to Look: guided attention inference network. 2018 IEEE/CVF conference on computer vision and pattern recognition (2018). 10.1109/CVPR.2018.00960 31180839

[35] Wang L Wu Z Karanam S Peng KC Singh RV Liu B Sharpen focus: learning with attention separability and consistency. 2019 IEEE/CVF international conference on computer vision (ICCV) (2019). 10.1109/iccv.2019.00060

[36] Ross AS Hughes MC Doshi-Velez F. Right for the right reasons: training differentiable models by constraining their explanations. Proceedings of the twenty-sixth international joint conference on artificial intelligence (2017). 10.24963/ijcai.2017/371

[37] Read the Docs. Welcome to segmentation models pytorch’s documentation! (2020). Available from: https://segmentation-modelspytorch.readthedocs.io/en/latest/ (Accessed June 30, 2022).

[38] Tan M Le QV. EfficientNet: Rethinking Model Scaling for Convolutional Neural Networks. arXiv: 1905.11946. (2019).

[39] Liang Y Li S Yan C Li M Jiang C. Explaining the black-box model: a survey of local interpretation methods for deep neural networks. Neurocomputing. (2021) 419:168–82. 10.1016/j.neucom.2020.08.011

[40] Hacker P Krestel R Grundmann S Naumann F. Explainable AI under contract and tort law: legal incentives and technical challenges. Artificial Intelligence Law. (2020) 28:415–39. 10.1007/s10506-020-09260-6

[41] Geirhos R Jacobsen JH Michaelis C Zemel R Brendel W Bethge M Shortcut learning in deep neural networks. Nat Mach Intell. (2020) 2:665–73. 10.1038/s42256-020-00257-z

[42] Granese F Romanelli M Gorla D Palamidessi C Piantanida P. DOCTOR: A Simple Method for Detecting Misclassification Errors. arXiv:2106.02395. (2021).

